# Community mental health literacy in Tshwane region 1: A quantitative study

**DOI:** 10.4102/sajpsychiatry.v28i0.1661

**Published:** 2022-03-24

**Authors:** Dumisile Madlala, Pierre M. Joubert, Andries Masenge

**Affiliations:** 1 Department of Health, Faculty of Health Sciences, Tshwane Mental Health District Services, Tshwane, South Africa; 2 Department of Psychiatry, Faculty of Health Science, University of Pretoria, Pretoria, South Africa; 3 Department of Statistics, Natural and Agricultural Science, University of Pretoria, Pretoria, South Africa

**Keywords:** community, mental health literacy, mental health, depression, schizophrenia, GAD

## Abstract

**Background:**

Although mental health literacy is a major determining factor of mental health outcomes and functional capacity of individuals, there is dearth of research on the issue in South Africa.

**Aim:**

To assess the literacy of three mental disorders, namely major depressive disorder (MDD), schizophrenia and generalised anxiety disorder (GAD) and to compare the resultant assumed literacy level between urban and townships participants.

**Setting:**

Five clinics of region 1 in Tshwane, South Africa.

**Method:**

A cross-sectional descriptive study was performed between November 2019 and January 2020. A total of 385 questionnaires were distributed equally in all five clinics. By means of questions about three fictive cases with clinical pictures indicative of MDD, schizophrenia and GAD the following were assessed: recognising a mental disorder, identifying the cause and knowledge about what would help best.

**Results:**

The majority of participants (67.3%) recognised the clinical picture indicative of schizophrenia as a mental disorder, almost half of the participants (49.9%) recognised the clinical picture indicative of MDD as a mental disorder, whilst just more than one third (36.3%) of participants recognised the clinical picture GAD as a mental disorder. Concerning the causes for the clinical pictures, most participants indicated that stress was the cause for MDD and GAD (77.4% and 68.1%, respectively), whilst indicating that biological or psychological (59.5%) causes are relevant to the clinical picture indicative of schizophrenia symptoms. Fewer participants indicated supernatural causes for any of the clinical case (MDD: 2.6%; schizophrenia 15.3%; GAD 4.2%). Most participants chose professional help as the best option for all three cases (MDD 81.3%, schizophrenia 82.2%, GAD 66.1%). The indicators for health literacy in this study show that urban participants had better knowledge than township participants across all questions about the cases.

**Conclusion:**

Overall, the study indicated a variable knowledge regarding the three mental disorders in region 1 of Tshwane and variable literacy levels in townships compared with urban settings. The results indicate that awareness campaigns should focus on the deficient areas.

## Introduction

Mental health is more than just lack of a mental illness. It is defined as mental functioning that enables one to recognise one’s abilities, cope with the normal stresses of life, work productively and make a positive contribution to one’s community.^1^ Mental health literacy is defined as knowledge and beliefs about mental disorders that aid in the recognition, management and prevention of mental disorders.^2^ It includes seven components: (1) the ability to recognise specific disorders, (2) knowing how to seek mental health-related information, (3) knowledge on causes, (4) knowledge of risks factors for mental disorders, (5) knowledge on self-treatments available, (6) knowledge on professional help available and (7) attitudes that promote recognition and appropriate help seeking.^2^ Lack of knowledge in the different aspects of mental health literacy has a major impact in the total outcome of mental health status of individuals and their communities.^3^ Patients’ responses are motivated not only by the availability of services but also by their knowledge about the services and how to access the services.^4^ The ability to recognise specific disorders and knowledge on how to seek mental health information prompt early help-seeking behaviours and early attendance with a relevant mental health team.^5^ This in turn promotes the early identification of mental disorders and the treatment thereof, which in turn promotes the mental health of that particular community.

As there is a correlation between mental health literacy and mental health outcomes,^4,5^ it is not surprising that this construct has been a subject of extensive research in many countries.

Studies have shown variable outcomes depending on the region researched. For example, Australian surveys indicate a steady improvement of mental health literacy over the years,^6,7^ while studies in some parts of Europe and Asia found lower levels of literacy than those of Australia.^8,9,10,11,12,13^

African studies revealed poor mental health literacy regarding the recognition, causes and professional treatment of mental disorders and consequently, negative attitudes towards mentally ill individuals.^14^ Studies in Ethiopia and Nigeria,^15,16^ for example, determined that respondents could only identify overt psychotic symptoms as signs of mental disorders. This means that common psychiatric disorders such as depressive disorders and anxiety disorders were not recognised. Furthermore, mental disorders were mainly attributed to supernatural causes rather than biological causes.^17^

South Africa is a middle-income country in Africa with an estimated population of 57 million.^18^ The country is fraught with many societal problems posing risk factors for mental disorders (e.g. violence and racial discrimination).^19^ Nonetheless, little has been published on mental health literacy in South Africa. The available studies indicate a lack of mental health literacy. One study reports that respondents mostly attributed the symptoms of mental disorders to stress, while ignoring biogenetic factors.^20^ A study involving families with relatives suffering from schizophrenia, reports that mental illness was often attributed to supernatural causes such as bewitchment.^21^ Furthermore, South African studies also indicate high prevalence rates of stigmatising mental disorders.^22,23,24^ Regarding stigma, Kakuma et al.^25^ reported on awareness campaigns to improve mental health literacy, but the impact of such campaigns remain unclear. Reports on the state of mental health systems in South Africa indicate the following: variation in service provision between the different provinces,^26^ treatment gaps, about 70% of mentally ill people do not receive treatment,^27^ a limited scope of decentralised community-based care,^28^ poor mental health policy implementation,^28^ a lack of uniform indicators for mental health information systems,^28^ a lack of involvement of mental healthcare users in mental health policy development.^28^

Considering all these facts, one questions how much South Africans know about mental disorders. Consequently, this study aimed to describe the mental health literacy of three common psychiatric disorders, namely generalised anxiety disorder, schizophrenia and major depressive disorder (MDD) in a region in South Africa. According to the South African health and stress study (SASH), anxiety disorders were found to be the most common with a prevalence of 13%. Mood disorders were found to have a prevalence of 9%.^27^ The prevalence of schizophrenia, a psychotic disorder, was not assessed in the SASH study but has a prevalence of about 1% worldwide.^29^

## Methods

The study was performed in region 1 of Tshwane, in the Gauteng province of South Africa. Gauteng is one of nine provinces in South Africa. Tshwane is one of five metropolitan municipalities in Gauteng province. Tshwane is divided into seven demographic regions, namely Regions 1–7. Tshwane has about 2.2 million people, with region 1 having about 811 570 people.^30^

Tshwane has 61 clinics that serve about 250 people daily in each clinic. This study was performed in five of these clinics, namely Tlamelong Clinic, Kgabo Clinic, Phedesong 4 Clinic, Soshanguve Clinic 3 and Pretoria North Clinic. The latter is the only urban clinic. The others are township clinics. The clinics were randomly selected.

### Study design and sampling

This quantitative, cross-sectional, descriptive study was carried out between November 2019 and January 2020. A total of 385 participants were recruited by convenience sampling. This amounted to 77 participants per clinic.

The sample size was informed by statistics: A sample of at least 384 is needed for a 95% confidence and 5% margin of error for a population of more than 1 000 000.^31^ The inclusion criteria to participate were: men or women from all race and ethnic groups aged 18 years and older and exclusion criteria were: people under 18 years of age and people unable to read or write.

Service users waiting to see health practitioners, as well as their accompanying family members were approached to take part in the study and briefed about it. This was done in English, isiZulu or Setswana, as needed, by the first researcher, who can speak all three languages. The fictive cases, questions about the cases (see Box 1), were also translated into isiZulu and Setswana. In the experience of the first researcher, these three languages are commonly used in the clinics where the study was performed. Participants signed the consent form and after that were given the research cases (Appendix 1) and questionnaire.

BOX 1 Questions about the cases.

1. What type of illness do you think the persons are suffering from?
  - Physical illness
  - Mental illness*
  - None of the above
  - I don’t know
2. What do you think caused the persons’ suffering?
  - Stress
  - Punishment from God/ancestors/bewitchment
  - Brain/genetic/psychological*
  - None of the above
3. What help is best for the persons?
  - Exercise/diet
  - Visit traditional healer
  - Medication/psychotherapy/counselling*

*, asterisk indicates that it is the correct answer.

### The questionnaire

The questionnaire was divided into two sections, namely sections A and B. Section A contained the demographic data and section B contained the questions to assess the mental health literacy. In order to assess mental health literacy, respondents were presented with three case studies that, respectively, meet the DSM-5^29^ criteria for MDD (‘Betty’), schizophrenia (‘Muzzy’) and generalised anxiety disorder (‘Lucy’). These disorders were selected because anxiety and mood disorders are common in South Africa.^27^

Although the global prevalence of schizophrenia is less, it is nonetheless relatively common.^29^ Although this is the case, symptoms of schizophrenia seem to be more likely to be identified as belonging to a mental disorder, especially in African countries.^14,15^ Hence, schizophrenia was included.

This study asked participants to choose one answer amongst various options for three questions about the fictive cases: (1) What type of illness do you think the persons are suffering from? (2) What do you think caused the persons’ suffering? (3) What help is best for the persons? (See Box 1: Questions about the Cases and Appendix 1). Thus, three specific aspects of mental health literacy were targeted, based on the work of Jorm et al.^2^ Like the fictive cases, the questions were also translated in Setswana and isiZulu.

### Ethical considerations

The study was approved by the Tshwane District Research Committee and Research Ethics Committee of the Faculty of Health Sciences, University of Pretoria. Ethics Reference No.: 543/2019.

### Statistical analysis

Frequency tables (counts and percentages) were performed for demographic variables (gender, employment status, marital status and level of education) and participants chosen answer for the type of problem, cause of the problem and best help for the problem. Cross tabulation was performed between the township clinics and the urban clinic where participants were enrolled and evaluated. Finally, the z test of proportion was conducted to test if the clinics’ participants differed proportionally in their response. The statistical significance was set at *p* < 0.05.

## Results

The demographic results are displayed in Table 1. A total of 77 questionnaires were distributed in each of the five clinics. The sum total of all questionnaires for all the clinics was 385. Each of the respondents was required to give responses to all three cases, namely depression, schizophrenia and generalised anxiety disorder (GAD). The descriptive statistical results obtained from participants’ chosen answers are displayed in Tables 2–4. From these tables it is evident that not all participants responded to all cases and all questions about the cases. The descriptive statistics comparing urban and rural outcomes are displayed in Table 5.

**TABLE 1:** Demographic data (*n* = 385).

Variables	*n*	%
**Gender**		
Male	86	22.3
Female	299	77.7
**Age**		
18–25	50	12.9
26–60	335	87.1
**Marital status**		
Single	253	65.7
Married	113	29.3
Divorce	10	2.6
Widowed	9	2.4
**Employment status**		
Full time employed	85	22.1
Part time employed	46	11.9
Unemployed	225	58.6
Self employed	29	7.4
**Education level**		
Never schooled	9	2.3
Special school	6	1.5
Grade 1–7	25	6.7
Grade 8–12	175	45.4
Post grade 12	170	44.1

**TABLE 2:** Participants’ responses with regard to recognition of mental disorder.

Options	MDD case of Betty *n* = 381		Schizophrenia case of Muzzy *n* = 373		GAD case of Lucy *n* = 369	
	*n*	%	*n*	%	*n*	%
Physical problem	142	37.3	77	20.6	173	46.9
Mental disorder	190	49.9	251	67.3	134	36.3
None of the above	29	7.6	22	5.9	36	9.8
I don’t know	20	5.2	23	6.2	26	7.0

MDD, major depressive disorder; GAD, generalised anxiety disorder.

**TABLE 3:** Participants’ responses with regard to cause of suffering.

Options	MDD Case of Betty(*n* = 350)		Schizophrenia Case of Muzzy (*n* = 353)		GAD Case of Lucy(*n* = 367)	
	*n*	%	*n*	%	*n*	%
Stress	271	77.4	81	22.9	253	68.1
Punishment from God/ancestors/bewitchment	9	2.6	54	15.3	15	4.2
Brain/genetic/psychological	64	18.3	210	59.5	81	22.7
None of the above	6	1.7	8	2.3	18	5
I don’t know	0	0	0	0	0	0

MDD, major depressive disorder; GAD, generalised anxiety disorder.

**TABLE 4:** Participants’ responses regarding best form of treatment.

Options	MDD case of Betty (*n* = 364)		Schizophrenia case of Muzzy (*n* = 361)		GAD case of Lucy(*n* = 357)	
	*n*	%	*n*	%	*n*	%
Exercise/diet	48	13.2	17	4.7	92	25.8
Visit a traditional healer	16	4.4	45	12.5	19	5.3
Medication/therapy/counseling	296	81.3	296	82.2	236	66.1
None of the above	4	1.1	3	0.8	10	2.8
I don’t know	0	0	0	0	0	0

MDD, major depressive disorder; GAD, generalised anxiety disorder.

**TABLE 5:** Comparison between an urban clinic and township clinics.

Variable	Township clinic: Correct answer		Urban clinic: Correct answer		*p*-value at 95% CI
	*n*	%	*n*	%	
**Recognising mental illness**					
MDD	142	46.7	48	62.3	0.015
Schizophrenia	198	68.4	53	70.7	0.486
GAD	104	35.3	30	40.5	0.399
**Cause of suffering**					
MDD	40	14.3	24	34.3	0.043
Schizophrenia	164	58.4	46	63.9	0.878
GAD	64	22.3	17	23.4	0.337
**Best form of treatment**					
MDD	235	81.3	61	81.3	0.997
Schizophrenia	235	81.9	61	82.4	0.913
GAD	192	67.8	44	59.5	0.231

CI, confidence interval; MDD, major depressive disorder; GAD, generalised anxiety disorder.

### On recognising a mental disorder

See Table 2 for the results of participants’ chosen answers to questions regarding the ability to identify a mental disorder. In all cases the correct choice is mental illness. Out of 381 participants who responded, the chosen answer for the case about MDD was a physical illness by 142 (37.3%), a mental illness by 190 (49.9%), neither a physical nor a mental illness by 29 (7.6%), while 20 (5.2%) did not know. Out of 373 participants who responded, the chosen answer for the case about schizophrenia was a physical illness by 77 (20.6%), a mental illness by 251 (67.3%), neither physical nor mental illness by 22 (5.9%), while 23 (6.2%) did not know. Out of 369 participants who responded, the chosen answer for the case of GAD was a physical illness by 173 (46.9%), a mental illness by 134 (36.3%), neither a physical nor a mental illness by 36 (9.8%), while 26 (7.0%) did not know.

### On the cause of suffering

See Table 3 for the results of participants’ chosen answers to the question regarding the ability to identify the cause of the suffering in the cases. In all cases the correct choice is either brain or genetic or psychological. Out of 350 participants who responded, the chosen answer about the cause of suffering in the case about MDD was stress by 271 (77.4%), punishment from God, ancestors or bewitchment by 9 (2.6%), brain, genetic or psychological by 64 (18.3%), none of the given options by 6 (1.7%), while no one chose, ‘I don’t know’. Out of 353 participants who responded, the chosen answer for the cause of suffering in the case about schizophrenia was stress by 81 (22.9%), punishment from God, ancestors or bewitchment by 54 (15.3%), brain, genetic or psychological by 210 (59.5%), none of the given options by 8 (2.3%), while no one chose, ‘I don’t know’. Out of 367 participants who responded, the chosen answer about the cause of suffering in the case about GAD was ‘stress’ by 253 (68.1%), ‘punishment from God/ancestors/bewitchment’ by 15 (4.2%), ‘brain/genetic/psychological’ by 81 (22.7%), none of the given options by 18 (5%), while no one chose, ‘I don’t know’.

### On the best form of treatment

Out of 364 participants who responded (see Table 4), the chosen answer for the best form of treatment in the case about MDD was exercise/diet by 48 (13.2%), a visit to a traditional healer by 16 (4.4%), medication/therapy by 296 (81.3%), none of the options by 4 (1.1%), whilst no one chose, ‘I don’t know’. Out of 361 participants who responded, the chosen answer for the best form of treatment in the case about schizophrenia was ‘exercise/diet’ by 17 (4.7%), ‘a visit to a traditional healer’ by 45 (12.5%), ‘medication/therapy’ by 296 (82.2%), none of the options by 3 (0.8%), whilst no one chose, ‘I don’t know’. Out of 357 participants who responded, the chosen answer for the best form of treatment in the case about GAD was ‘exercise/diet’ by 92 (25.8%), a visit to a traditional healer by 19 (5.3%), ‘medication/therapy’ by 236 (66.1%), none of the options by 10 (2.8%), whilst no one chose, ‘I don’t know’.

### On comparing the results between an urban clinic and township clinics

Referring to Table 5, here follow the comparisons between the descriptive statistics of township and an urban clinic regarding the correct chosen answers for the different case-related questions. Firstly, correctly choosing ‘mental illness’ as answers to the question about the kind of illness the fictive cases portrayed; for township clinics versus urban clinic: MDD, 142 (46.7%):48 (62.3%) (*p* = 0.015 at 95% CI [confidence interval]); schizophrenia, 198 (68.4%): 53 (70.7%) (*p* = 0.486 at 95% CI); GAD, 104 (35.3):30 (40.5%) (*p* = 0.399 at 95% CI). Secondly, correctly choosing ‘brain/genetic/psychological’ as answer to the question about the cause of suffering; township clinics versus urban clinic: MDD, 40 (14.3%):24 (34.3%) (*p* = 0.043 at 95% CI); schizophrenia, 164 (58.4%):46 (63.9%) (*p* = 0.878 at 95% CI); GAD, 64 (22.3%):17 (23.4%) (*p* = 0.337 at 95% CI). Thirdly, correctly choosing ‘medication/psychotherapy/counselling’ as the answer to the question about the best form of treatment; township clinics versus urban clinic: MDD, 235 (81.3%):61 (81.3%) (*p* = 0.997 at 95% CI); schizophrenia, 235 (81.9%):61 (82.4%) (*p* = 0.913 at 95% CI); GAD, 192 (67.8%):44 (59.5%) (*p* = 0.231 at 95% CI). Thus, there are only two parameters where there is a significant difference between the urban clinic and township clinics regarding the correct choice of answer: Choosing MDD as a mental illness and choosing the cause of suffering to be ‘brain/genetic/psychological’; in both cases the urban clinic did better.

## Discussion

This study investigated the mental health literacy of participants from clinics in region 1 of the Tshwane District. Mental health literacy was assessed by using Jorm’s^2^ definition, namely recognition of a mental illness, knowledge of causation and knowledge of the best help available. Participants were requested to complete a questionnaire after having read three fictive cases that, respectively, indicated MDD, schizophrenia and GAD. Based on the answers chosen by about half of the participants, the mental illness, MDD, was readily recognised as a mental illness after reading the fictive case. Participants from the urban clinic chose mental illness significantly more frequently than those from the township clinics. That being so, the overall results are encouraging, because recognising mental illness is associated with appropriate help seeking.^31,32,33,34^ Nonetheless, about half of all the participants did not recognise the mental illness. The latter group included those who chose physical illness as the answer (more than one third) or (much fewer) neither a mental nor physical illness, or chose that they did not know. Two implications are immediately apparent: (1) clinicians should be aware that MDD (perhaps especially in township areas) may present as a physical illness (2) more community-based psychoeducation is needed.

For the MDD case, only a minority (18.3%) chose the correct answer to the question about the cause of suffering, namely ‘brain/genetic/psychological’, whilst an overwhelming majority (77.4%) chose ‘stress’. Since so many participants chose physical illness as the explanation for the symptoms of the MDD case, one wonders whether participants might have considered stress as a cause of physical illness (and mental illness), which would not be incorrect. Nonetheless, urban participants did significantly better than the township participants and this finding is consistent with other studies.^35,36,37^ It also indicates that people consider stress to be the cause of MDD and GAD.^38,39,40^ Identifying stress as the cause, is associated with seeking lay help rather than professional help.^39^ Unfortunately, a very small portion (2.6%), chose ‘punishment from God/ancestor/bewitchment’. This finding also supports continued community-based psychoeducation.

When it comes to the best treatment, the overwhelming majority chose ‘medication/therapy’. As most participants chose either mental illness or physical illness and since for both the choice ‘medication/therapy’ would be the correct choice, the outcome is to be expected. For this last question, urban and township participants faired equally well, perhaps further indicating that ‘medication/therapy’ was chosen for mental and physical illnesses. Thus, this answer too needs revision for future use, so as to clearly distinguish between the management of mental and physical illness. A sizable minority (13.2%) choose ‘diet/exercise’ and, much fewer, traditional healing as the best treatment option. Taken together, the findings about the MDD case calls for more community-based education.

Whilst about half of participants recognised the MDD case as a mental illness, most (67.3%) recognised the schizophrenia case a mental illness. with little difference between urban and township participants. Participants in this study did better than in a previous study.^21^

Nonetheless, a sizable portion (20.6%) chose physical illness, with much fewer choosing the case being neither a mental nor physical illness (5.9%) or that they don’t know (6.2%). The findings are in line with previous studies indicating that schizophrenia is more readily recognisable as a mental illness than MDD,^24,41,42^ and, by extension, GAD (which will be discussed here). Thus, it seems less likely that people will confuse schizophrenia with a physical illness and thus, seek appropriate help. It is possible that community-based education and lay publications give more attention to schizophrenia than MDD and GAD. However, it is also possible that the dramatic nature of psychotic symptoms readily alert community members to a mental illness.

Contrary to the MDD case (and the following GAD case) most participants (59.5%) chose the correct answer about the cause of suffering, with urban participants doing somewhat better. It is possible that most participants considered ‘brain/genetic/psychological’ to be the (obvious) cause, thereby simply excluding ‘stress’ from the picture, but yet, a sizable minority (22.9%) chose ‘stress’. A sizable minority (15.3%) also chose ‘punishment from God/ancestor/bewitchment’ as the cause of suffering is worrying. This answer was chosen with greater frequency than for the MDD (2.6%) and GAD (4.2%) cases. Despite that, fewer participants in this study chose the supernatural as cause for MDD and GAD^43^ and schizophrenia^44^ than in other studies. The implications are that, although schizophrenia is readily recognised as mental disorder, a sizable minority may consider the cause of suffering to be supernatural. That in turn may have an effect on seeking appropriate help and the way a psychotically ill person is viewed and handled by the community, for example, searching out spiritualists and traditional healers.^38,39,40^ The need for community-based psychoeducation is again indicated.

Whilst participants recognised mental illness regarding the MDD and schizophrenia cases, they had, by inference, difficulty doing so for GAD: physical illness (46.9%) was more frequently chosen than mental illness (36.3%), whilst a few chose neither (9.8%) or did not know (7.0%).

Participants from both urban and township clinics did poorly with GAD. Possible reasons for this are lack of community-based education, fears (if one should recognise oneself in the case) of being seen as weak or stigmatisation.^33^ This outcome may indicate that few people with GAD will seek appropriate help.

Participants mostly chose stress as the cause of suffering (68.1%), with a sizable minority choosing ‘brain/genetic/psychological’ (22.7%) and a smaller minority (5.0%) choosing neither. As for MDD and schizophrenia, participants again chose ‘medication/therapy’ as best treatment. However, many participants (25.8%) chose ‘exercise/diet’ as the best treatment, more frequently than for MDD and schizophrenia, perhaps reflecting the perceptions of stress as the cause. Like for MDD, this may be because many chose the GAD case to reflect physical illness, and thus, medication would be a logical treatment choice.

## Limitations

The study used only three mental illnesses, targeted very specific aspects of mental health literacy and sourced participants from very specific areas in Gauteng. Furthermore, doing the study at clinics may have selected participants with biases that may be different from people who never use or need to use medical clinics. Thus, the outcomes of the study should not readily be generalised. Nonetheless, the study gives a glimpse into a poorly researched area. In retrospect it is possible that at least some participants found the difference between the options, ‘stress’ and ‘brain/genetic/psychological’ confusing because stress and psychological are closely related. As pointed out here, medication would be the treatment choice for both mental illness and physical illness. Consequently, the study cannot claim that when participants chose the treatment option, ‘medication’, it was for a mental illness. The study mostly used descriptive statistics, rather than analyses of significance when comparing outcomes related to the three mental illnesses. Nonetheless, at face value, the descriptive statistics are informative.

## Conclusion

Participants in this study correctly recognised and identified causation and the best treatment for schizophrenia, followed by MDD, and then GAD. Participants from an urban area did somewhat better than those from township areas. The study indicates that, especially for MDD and GAD, mental illness is confused with physical illness. Furthermore, a minority attributed clinical features of a mental illness as punishment from God. Altogether, there is a reason to be optimistic and concerned.

## Appendix 1

### The Cases

#### Betty

Betty is a 34 year old married female with two children, she presents with 4 weeks history of the following symptoms:

1. She has low mood every day and most of the day.
2. She lacks interest and does not enjoy things anymore most of the time, nearly every day.
3. She feels sad, empty and hopeless.
4. She is not sleeping well.
5. She is very slow.
6. She is always tired.
7. She feels small, has low self-esteem and feels worthless and guilty about a lot of things.
8. She cannot think clearly and concentrate.
9. She thinks it is better to die.

As a result she is no longer able to take care of her family

She is physically health and does not use drugs.

Tick what you think is correct

#### Muzzy

Muzzy is 25 years of age. He is not married. He dropped out of university in his first year about 3 years ago. Since then he has often been hearing a voice that tells him what to do. He then also started talking in a way that does not make sense. He became very aggressive towards his uncle, and accused his uncle of planting wires inside his stomach. He still believes that his uncle did it. At present he does not like to mix with others and prefers to do nothing.

He is physically healthy and does not use drugs.

#### Lucy

Lucy is a 37 year old female. She experiences excessive worry and she is always anxious. She finds it difficult to control the worries and it affects her ability to do her chores. She also experiences the following symptoms:

1. She is restless.
2. She becomes tired easily.
3. She cannot concentrate.
4. She is irritable.
5. She has muscle tension.
6. She has problems with sleeping.

She physically healthy and does not use drugs.
